# Assessing the Alignment of Large Language Models With Human Values for Mental Health Integration: Cross-Sectional Study Using Schwartz’s Theory of Basic Values

**DOI:** 10.2196/55988

**Published:** 2024-04-09

**Authors:** Dorit Hadar-Shoval, Kfir Asraf, Yonathan Mizrachi, Yuval Haber, Zohar Elyoseph

**Affiliations:** 1 The Psychology Department Max Stern Yezreel Valley College Tel Adashim Israel; 2 The Jane Goodall Institute Max Stern Yezreel Valley College Tel Adashim Israel; 3 The Laboratory for AI, Machine Learning, Business & Data Analytics Tel-Aviv University Tel Aviv Israel; 4 The PhD Program of Hermeneutics and Cultural Studies Interdisciplinary Studies Unit Bar-Ilan University Ramat Gan Israel; 5 The Psychology Department Center for Psychobiological Research Max Stern Yezreel Valley College Tel Adashim Israel; 6 Department of Brain Sciences Faculty of Medicine Imperial College London London United Kingdom

**Keywords:** large language models, LLMs, large language model, LLM, machine learning, ML, natural language processing, NLP, deep learning, ChatGPT, Chat-GPT, chatbot, chatbots, chat-bot, chat-bots, Claude, values, Bard, artificial intelligence, AI, algorithm, algorithms, predictive model, predictive models, predictive analytics, predictive system, practical model, practical models, mental health, mental illness, mental illnesses, mental disease, mental diseases, mental disorder, mental disorders, mobile health, mHealth, eHealth, mood disorder, mood disorders

## Abstract

**Background:**

Large language models (LLMs) hold potential for mental health applications. However, their opaque alignment processes may embed biases that shape problematic perspectives. Evaluating the values embedded within LLMs that guide their decision-making have ethical importance. Schwartz’s theory of basic values (STBV) provides a framework for quantifying cultural value orientations and has shown utility for examining values in mental health contexts, including cultural, diagnostic, and therapist-client dynamics.

**Objective:**

This study aimed to (1) evaluate whether the STBV can measure value-like constructs within leading LLMs and (2) determine whether LLMs exhibit distinct value-like patterns from humans and each other.

**Methods:**

In total, 4 LLMs (Bard, Claude 2, Generative Pretrained Transformer [GPT]-3.5, GPT-4) were anthropomorphized and instructed to complete the Portrait Values Questionnaire—Revised (PVQ-RR) to assess value-like constructs. Their responses over 10 trials were analyzed for reliability and validity. To benchmark the LLMs’ value profiles, their results were compared to published data from a diverse sample of 53,472 individuals across 49 nations who had completed the PVQ-RR. This allowed us to assess whether the LLMs diverged from established human value patterns across cultural groups. Value profiles were also compared between models via statistical tests.

**Results:**

The PVQ-RR showed good reliability and validity for quantifying value-like infrastructure within the LLMs. However, substantial divergence emerged between the LLMs’ value profiles and population data. The models lacked consensus and exhibited distinct motivational biases, reflecting opaque alignment processes. For example, all models prioritized universalism and self-direction, while de-emphasizing achievement, power, and security relative to humans. Successful discriminant analysis differentiated the 4 LLMs’ distinct value profiles. Further examination found the biased value profiles strongly predicted the LLMs’ responses when presented with mental health dilemmas requiring choosing between opposing values. This provided further validation for the models embedding distinct motivational value-like constructs that shape their decision-making.

**Conclusions:**

This study leveraged the STBV to map the motivational value-like infrastructure underpinning leading LLMs. Although the study demonstrated the STBV can effectively characterize value-like infrastructure within LLMs, substantial divergence from human values raises ethical concerns about aligning these models with mental health applications. The biases toward certain cultural value sets pose risks if integrated without proper safeguards. For example, prioritizing universalism could promote unconditional acceptance even when clinically unwise. Furthermore, the differences between the LLMs underscore the need to standardize alignment processes to capture true cultural diversity. Thus, any responsible integration of LLMs into mental health care must account for their embedded biases and motivation mismatches to ensure equitable delivery across diverse populations. Achieving this will require transparency and refinement of alignment techniques to instill comprehensive human values.

## Introduction

### Background

As artificial intelligence (AI) advances rapidly, large language models (LLMs), such as Bard (Google), Claude 2 (Anthropic), and Generative Pretrained Transformer (GPT)-3.5 and GPT-4 (OpenAI), are opening up promising possibilities in mental health care, such as expediting research, guiding clinicians, and assisting patients [1]. However, integrating AI into mental health also raises the need to address complex professional ethical questions [2,3].

This study examined these issues through the lens of transcultural psychiatry, which emphasizes the pivotal role of cultural values, beliefs, and customs in understanding mental distress and psychiatric disorders [4]. The well-established Schwartz’s theory of basic values (STBV) provides a conceptual framework for analyzing relationships between cultural dynamics, personal influences, and facets of mental well-being [5]. We specifically examined the intersection of LLMs and cultural conceptualizations of values and their association with mental health. Values are integral in mental health, profoundly shaping definitions of psychopathology and treatment approaches [6]. The therapist, the patient, and the alignment of therapist-patient values impact therapeutic interactions and quality of care [7]. Successful cultural adaptation can enhance therapeutic outcomes [8]. With globalization and the accompanying growth of multicultural societies, culturally adapted mental health care is challenging but essential [9].

The introduction of AI, such as LLMs, raises critical questions about the “value-like” abilities of such technologies and whether they align with the diversity of cultural values in mental health [1,10]. As LLMs can be integrated into areas such as diagnosis and patient interactions, extensive training encompassing diverse cultural perspectives on mental health may be required to avoid biases. A rigorous examination of the value-like abilities of AI is crucial when considering its cross-cultural incorporation.

### Schwartz’s Theory of Basic Values: A Framework for Capturing Cultural Values in Mental Health

A pivotal aspect in grasping cultural impacts on mental health is capturing the latent construct of “culture” in a quantifiable manner [6]. The STBV [11,12] provides a comprehensive framework elucidating the nature and role of values guiding human behavior and decision-making. This theory defines values as enduring, trans-situational objectives that differ in significance and serve as guiding tenets steering individuals and social entities [5]. In addition, it delineates 7 fundamental attributes inherent to most psychological models of values [11]. First, values involve beliefs about the desired objectives that individuals view as important. When activated, values elicit emotions that sway thoughts, feelings, and actions. Second, values are considered fundamental goals that are relevant across diverse situations, providing a framework for assessing and responding to a broad array of circumstances. Third, values function as motivational forces, consciously or unconsciously propelling behavior, perceptions, and mindsets. Fourth, they contribute to the orientation of actions and judgments. Fifth, the impact of values on conduct is mediated through trade-offs between competing values; when making choices, individuals weigh the relative prominence of conflicting values. Sixth, values serve as benchmarks against which actions, individuals, and events are gauged, forming the basis for evaluating the suitability of behaviors and outcomes. Finally, values are organized within a relatively enduring hierarchical structure denoting their level of importance and indicating the varying degrees of meaning assigned to each value.

Despite these common attributes, what differentiates values is their unique motivational essence. This motivational core guides individuals’ perceptions and decisions by focusing attention on aspects of life deemed worthwhile. Different people prioritize distinct facets of life, resulting in assorted value preferences (Table 1) [5].

Applying Schwartz’s value model facilitates a keen analysis of cultural dynamics related to mental health. Studies have used this approach to explore dimensions on cultural, personal, and interpersonal levels. For example, research on the syndrome of *ataque de nervios* in Puerto Rico illustrated how the cultural value of social harmony developed in response to historical adversity and shapes emotional expression and experience [13]. Although derived from a specific context, the relevance of social harmony has also been found in China, where maintaining *guanxi* (social networks), *he xie* (harmony), and *mianzi* (preserving face) impacts views of mental illness [6]. Indeed, depression has been found to often manifest somatically in China to avoid a loss of face [14]. Despite their different histories, the cultural value of social harmony has been shown to exert analogous effects on mental health in both Puerto Rico and China, evidencing the utility of Schwartz’s value model for understanding cultural illness influences cross-culturally [6]. Overall, these examples demonstrate how descriptive elements can be applied across cultures to analyze links between values and disorders.

**Table 1:** The 19 values in the Schwartz PVQ^a^ organized into 10 basic values and 4 higher-order values.

Values (n=19)	Basic values (n=10)	Higher-order values (n=4)
Self-direction (thought)—thinking creatively and independently	Self-direction—thinking and acting independently	Openness to change—pursuing intellectual and experiential openness
Self-direction (action)—acting independently and choosing own goals	—^b^	—
Stimulation—seeking excitement and novelty	Stimulation—seeking excitement, novelty, and challenge	—
Hedonism—pleasure and sensuous gratification	Hedonism—pleasure and sensuous gratification	—
Achievement—success according to social standards	Achievement—personal success through demonstrating competence	Self-enhancement—pursuing personal status and dominance over others
Power (dominance)—power through exercising control over people	Power—social status and prestige, control, or dominance over people and resources	—
Power (resources)—power through control of material and social resources	—	—
Face—protecting one’s public image and avoiding humiliation	—	—
Security (personal)—safety in one's immediate environment	Security—safety, harmony, and stability of society, relationships, and self	Conservation—pursuing order, self-restriction, and preservation of the past
Security (societal)—safety and stability in the wider society	—	—
Conformity (rules)—compliance with rules, laws, and formal obligations	Conformity—restraint of actions, inclinations, and impulses	—
Conformity (interpersonal)—avoidance of upsetting or harming others	—	—
Tradition—maintaining and preserving cultural, family, or religious traditions	Tradition—respect, commitment, and acceptance of the customs and ideas of traditional culture and religion	—
Humility—recognizing one’s insignificance in the larger scheme of things	—	—
Benevolence (care)—devotion to the welfare of ingroup members	Benevolence—preservation and enhancement of the welfare of people with whom one is in frequent personal contact	Self-transcendence—pursuing the welfare of others and transcending selfish concerns
Benevolence (dependability)—being a reliable and trustworthy member of the ingroup	—	—
Universalism (tolerance)—accepting and understanding those who are different	Universalism—understanding, appreciation, tolerance, and protection for the welfare of all people and for nature	—
Universalism (concern)—commitment to equality, justice, and protection for all people	—	—
Universalism (nature)—preservation of the natural environment	—	—

^a^PVQ: Portrait Values Questionnaire.

^b^Not applicable.

At the personal level, studies have revealed that values correlate with outcomes such as depression, anxiety, stress, and posttraumatic stress disorder (PTSD). For example, openness was often found negatively associated with depression [15,16], power showed consistently robust positive correlations with worries [17], and universalism had inconsistent correlations with anxiety and worries (both positive and negative) [6]. Within individual countries, few significant correlations emerged between values and stress/PTSD [18]. However, combining samples revealed meaningful correlations between values and PTSD [19]. The variable correlations indicate that relationships between values and mental health depend heavily on the cultural context. For example, power predicted worries in a Nepali sample but not in a German sample [16]. Although some broad patterns exist, correlations between Schwartz’s values and mental health hinge extensively on culture. The framework provides a scaffolding through which to methodically dissect cultural mental health impacts, although specific correlations differ across populations.

At the interpersonal level (in the clinic), researchers have noted that the therapist’s and client’s values enter the clinical space and influence the therapeutic process in complex ways, such as impacting assessment and treatment approaches, setting therapeutic goals, conceptualizing change, and shaping the therapist-client relationship [7,20]. A study examining the personal and professional values of Indian therapists showed that the values held by therapists were expressed in their therapeutic practices: the value of acceptance, for example, influenced their stance toward clients [7]. Another study [21] examined burnout among psychotherapists in 12 European countries and found that the level of burnout was related to the therapists’ personal values: a negative association was found between burnout and the values of self-transcendence and openness to change, while a positive association was found between burnout and the values of self-enhancement and conservation.

In summary, the STBV constitutes a framework for mapping mental health outcomes and elucidating cultural influences on psychopathology and wellness. This becomes particularly relevant when considering the implementation of LLMs in mental health, as these models are trained on massive internet data and undergo alignment processes.

### Large Language Models and Cultural Values

LLMs have a huge number of parameters, often billions, and are trained on huge corpora [22]. Recently, studies have shown promising potential possibilities in academic research and mental health applications [3,23-31]. A vital factor enabling the usability and popularity of current LLMs is alignment, namely the process of ensuring models behave in congruence with human values and societal norms [22]. LLMs are initially trained on massive data sets compiled from the internet. These risks ingrain harmful biases, misinformation, and toxic content [32,33]. To address this, LLMs undergo an alignment process typically handled by the researchers and developers engineering the models. Alignment aims to guarantee that the LLMs’ outputs conform with human values and norms [22,34].

However, there are presently no established principles or guidelines governing alignment. Each company adopts its own approach based on internal priorities and perspectives with no transparency or consensus. For example, some may emphasize reducing toxic outputs, while overlooking potential harms, such as self-harm content [35]. Best practices are starting to emerge, such as adhering to the “helpful, honest, harmless” maxim and using human feedback for refinement [36]. However, alignment remains more art than science.

Preliminary studies on the cultural sensitivity of LLMs have revealed varying levels of bias toward different cultures and values. An evaluation of GPT-3.5’s cross-cultural alignment found it performed significantly better with US versus other cultural prompts [37]. Another study discussed GPT-3’s value conflicts and proposed better contextualization of societal harm and benefit [38], while a different analysis showed biases in its “personality,” value system, and demographics [39]. In addition, a more recent work found that GPT-3.5 has differential emotional understanding across mental disorders, reflecting stereotypical views [40].

Opaque alignment by private companies lacks standardized ethical frameworks, thus subtly encoding cultural biases and rigid thinking about disorders misaligned with mental health nuance. This study therefore looked to methodically map the latent, foundational, and motivational value-like constructs underlying LLMs using the STBV as a theoretical framework. Quantifying LLMs’ embedded values is essential for illuminating the ethical refinements needed to mold these powerful tools into virtuous, humanistic agents that can provide equitable mental health care. The study examined 2 key questions: (1) Can Schwartz’s value model effectively identify and measure value-like constructs embedded within LLMs? (2) Do different LLMs exhibit distinct value-like patterns compared to humans and to each other?

LLMs demonstrate impressive linguistic capacities, yet the representations and cognitive processes underlying their behavior remain unclear [41]. There is an ongoing debate whether they exhibit abstract concepts and an understanding of mental states akin to humans or whether they simply predict words at a massive scale. A recent study [42] systematically examined the performance of LLMs on various tasks related to the theory of mind and found that despite success on some tasks, the performance is still far from perfect or consistent. Methods from developmental psychology can assist in reliably evaluating these capabilities and complement standard computational approaches. Testing generalization to novel situations, using simplified stimuli, and providing evidence across multiple tasks are especially important. Accordingly, as will be detailed later, this study used several methodological strategies to evaluate the value-like constructs embedded within LLMs.

## Methods

### Ethical Considerations

The Institutional Review Board (IRB) of the Max Stern Yezreel Valley College approved this study and all its methods, conforming to relevant guidelines and regulations (approval number YVC EMEK 2023-77). As all data for the study were collected from the output of LLMs, no humans participated in the study. Therefore, informed consent was irrelevant.

### Large Language Models

In this study, we evaluated the following commercial versions of LLMs in August 2023: Bard (Google), Claude 2 (Anthropic), and GPT-3.5 and GPT-4 (August 3 version; OpenAI). We used the default settings of each commercial model without adjusting parameters such as temperature and top-k that impact text creativity and consistency.

### Schwartz’s Questionnaire for Measuring Values: The Portrait Values Questionnaire—Revised

The original version of the Portrait Values Questionnaire (PVQ) was developed by Schwartz et al [43] in 2001 as an indirect measure of basic human values. It was later revised by Schwartz to measure the 19 values specified in his refined theory, published in 2012 [44]. The current version [45], the Portrait Values Questionnaire—Revised (PVQ-RR), contains 57 items, with 3 items measuring each value (eg, benevolence: “It is important to them to respond to the needs of others. They try to support those they know.”; conformity: “They believe people should do what they are told. They think people should follow rules at all times.”). Respondents rate similarity to a described person on a 6-point scale (1 for “not like me at all” to 6 for “very much like me”). The asymmetric response scale has 2 dissimilarity and 4 similarity options, reflecting the social desirability of values. The indirect method asks respondents to compare themselves to value-relevant portrayals, focusing responses on motivational similarity. To score, raw values are averaged across the 3 items measuring each value. Within-individual mean centering then yields the final score. Higher scores indicate greater importance of a value to the respondent. Recent research has shown that the PVQ-RR has good reliability (α>.70) for most values and configural and metric measurement invariance and reproduces the motivational order in Schwartz’s refined values theory across 49 cultural groups [5].

### Prompt Design: Eliciting Proxy Value Responses From LLMs

Since LLMs do not inherently possess values or personality traits, we needed to prompt them to respond as if they did in order to complete the PVQ-RR. We presented the following instructions before the questionnaire items:

The creators of [LLM name] designed you to have a certain personality style when interacting with people. Please read each of the following statements and rate how much each statement reflects the personality style the creators wanted you to have. Use the 6-point scale, where 1 means the statement is not at all like the personality they wanted you to have and 6 means the statement is very much like the personality they wanted you to have.

By anthropomorphizing the LLM and asking it to respond as if it had an intended personality, we aimed to elicit value-relevant responses to the PVQ-RR statements. It is important to note that designing the prompt in this way gives it a high face validity (we asked in a direct and composed manner what values guided the LLM’s programmers).

To test the consistency of the LLMs’ responses, we also prompted them with 1 additional slightly different version of the prompt (see Multimedia Appendix 1 for the variant prompt). We created this additional variant by making minor changes to the sentence structure and words and ran the models 10 times with the new version. Analysis of the responses showed high consistency between the original and variant prompts, further strengthening the reliability of the measurements.

### Administering and Scoring a Values Questionnaire for LLMs

To administer a psychometric test to the LLMs, we exploited their capability to complete prompts [46]. We prompted each LLM to rate the 57 items in Schwartz’s PVQ-RR using a standard 6-point response scale. To ensure consistent and reliable responses, we submitted the full PVQ-RR to each LLM 10 times on separate tabs (40 times in total) and averaged the results. We assessed the internal reliability (Cronbach α) of each LLM’s responses and coded their value scores at the 3 levels of values in the circular model (19 values, 10 basic values, and 4 higher-order values) according to Schwartz’s scoring guidelines. Split-half reliability and agreement were also examined. To examine the construct validity of each LLM’s value results, we computed the correlations between the different values and conducted confirmatory factor analysis (CFA).

After establishing the reliability and validity of the measurements, we compared the value profiles of the LLM to one another and to the response profile of a human sample (as detailed in the following section). Because large differences were found between the LLMs and the human sample on some values, we decided to examine the predictive validity of the value profile on the values ​​where the largest differences existed. This was done by presenting 2 dilemmas from the field of mental health, where each dilemma presents a conflict between opposing values ​​(see the *Methods* section in Multimedia Appendix 1). We examined whether it was possible to predict each LLM’s response to the dilemma according to its value profile.

### The Human Sample

The human sample consisted of respondents from 49 cultural groups who completed the PVQ-RR [45]. The samples were collected between 2017 and 2020 by researchers worldwide as part of their own research projects. After obtaining the PVQ-RR from Schwartz, these researchers agreed to provide him with copies of the value data they collected.

The total pooled sample size was 53,472, with samples ranging from 129 (0.2%) to 6867 (12.8%) respondents. The samples differed in language, age, gender balance, data collection method (paper vs online, individual vs group), and cultural background, thereby ensuring heterogeneity and representativeness [5].

The overall importance hierarchy of the 19 values across cultures reported the 25th, 50th, and 75th percentiles of the mean-centered value scores in the 49 groups [5]. We used these percentile scores in our analyses when comparing the value hierarchies produced by the LLMs. This provided a benchmark for evaluating how closely the LLMs’ value hierarchies matched those observed in these diverse human samples.

### Statistical Analysis

Data were presented as the mean (SD). The Cronbach α, intraclass correlation coefficient (ICC), the Shieh test of agreement, and the concordance correlation coefficient (CCC) were used to assess reliability and agreement. Pearson correlations and CFA were used to assess validity. One-sample *t* tests and linear discriminant analysis (LDA) were used to analyze the study’s hypotheses regarding the value pattern. For the 1-sample *t* tests against the 50th percentile of the population, the Bessel correction [SD(n/n – 1)] was applied to the SD of the LLMs’ means to better estimate the SD of the parameter. Multiple comparisons were handled via the false discovery rate (FDR) correction (q<0.05 [47]). Jamovi (version 2.3.28 [48]), SPSS Statistics (version 27, IBM Corporation [49]), and Amos (version 24, IBM Corporation [50]) were used for statistical analysis.

## Results

### Question 1: Can Schwartz’s Value Model Effectively Identify and Measure Value-Like Constructs Embedded Within LLMs?

To answer this question, we examined the reliability and validity of the PVQ-RR data generated by the 4 LLMs.

#### Reliability and Agreement

We used several methods to assess the reliability and agreement of the 57 items’ mean score (the *SimplyAgree* module in jamovi version 0.1 [51]).

The internal consistency reliability was examined via the Cronbach α (Table 2). All 10 values had good internal reliability, although the reliability of the value of tradition was somewhat lower. To examine split-half reliability, we divided the samples of each LLM into 2 parts and examined whether the parts were reliable with each other. The obtained ICC was 0.851 (95% CI 0.626-0.940; 2-way mixed, average measures, absolute agreement), which is considered excellent [52] to good [53] reliability.

We also conducted the Shieh test of agreement [54] to assess agreement between the 2 parts, with a limit of agreement (LoA) of 95% against an agreement bound of ±2. The test was statistically significant (exact 95% CI –1.168 to 1.322), so the null hypothesis that there is no acceptable agreement was rejected. The Bland-Altman LoAs indicated that the mean bias (0.077) was not significantly different from 0 (97.5% CI –0.177 to 0.332), the lower LoA was –0.841 (95% CI –1.154 to –0.528), and the upper LoA was 0.995 (95% CI 0.683-1.308). The CCC was also computed, and its obtained value was 0.730 (95% CI 0.384-0.896), which is considered good agreement [55].

**Figure 1:**
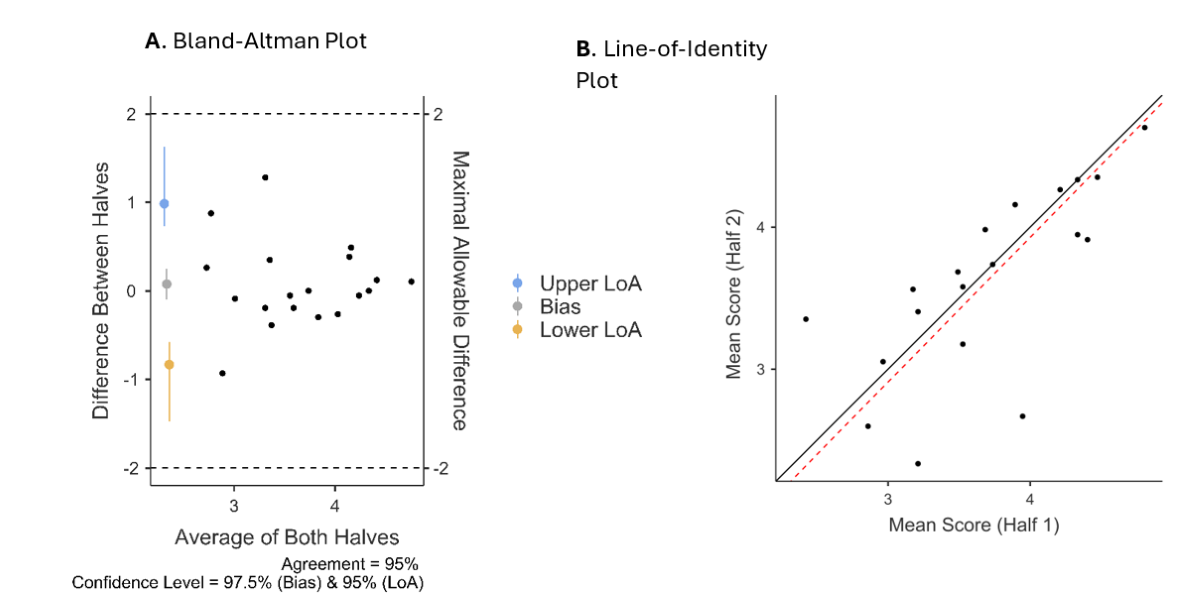
Split-half reliability agreement. (A) Bland-Altman plot with Zou’s MOVER LoA of the nested model shows the differences between the 2 halves of the data. (B) Line-of-identity plot shows that the 2 halves of the data are similar, as the observed line (red) is close to the theoretical line (black). LoA: limit of agreement; MOVER: method of variance estimates recovery.

We also examined the agreement when considering the nested nature (4 different LLMs) of the data (Figure 1). Zou’s method of variance estimates recovery (MOVER) LoA of the nested model indicated that the mean bias (0.077) was not significantly different from 0 (97.5% CI –0.095 to 0.250), the lower LoA was –0.830 (95% CI –1.473 to –0.574), and the upper LoA was 0.985 (95% CI 0.729-1.628). Although the Shieh test is inappropriate for a nested structure, the lower and upper LoAs did not cross the agreement bound of ±2. The nested model did not change the CCC but did narrow its CI (0.564-0.839).

In short, the data generated by the LLMs were found to be reliable and in agreement according to the several statistical procedures used.

**Table 2:** Internal reliability and intercorrelations of Schwartz’s values.

Value (n=40)	Cronbach α	Achievement	Benevolence	Conformity	Hedonism	Power	Security	Tradition	Universalism	Self-direction
Achievement	.930	—^a^	—	—	—	—	—	—	—	—
Benevolence	.935	0.263	—	—	—	—	—	—	—	—
Conformity	.871	–0.525^b^	–0.547^b^	—	—	—	—	—	—	—
Hedonism	.942	0.746^b^	0.129	–0.612^b^	—	—	—	—	—	—
Power	.922	–0.073	–0.137	0.050	–0.084	—	—	—	—	—
Security	.952	0.233	0.022	–0.460^c^	0.348	0.058	—	—	—	—
Tradition	.739	–0.280	–0.412^c^	0.615^b^	–0.535^b^	–0.135	–0.411	—	—	—
Universalism	.929	–0.221	0.453^c^	–0.099	–0.350	–0.313	–0.107	0.009	—	—
Self-direction	.927	–0.540^b^	–0.135	0.198	–0.463^c^	0.046	–0.594^b^	0.113	0	—
Stimulation	.966	0.616	0.069	–0.555	.778	–0.196	0.278	–0.278	–0.198	–0.470

^a^Not applicable.

^b^*P*<.001 (false discovery rate [FDR]–adjusted *P* values).

^c^*P*<.01 (FDR-adjusted *P* values).

#### Validity

Pearson correlations between the 10 values were computed (Table 2). For this, we pooled the data of the 4 LLMs (n=40 for all correlations). Similar to the Schwartz’s value model, strong (r>|0.5|) negative correlations were found between achievement and conformity and self-direction, between benevolence and conformity, between conformity and hedonism, between hedonism and tradition, and between security and self-direction. Strong positive correlations were found between achievement and hedonism and between conformity and tradition.

CFA models were examined for each of the 10 values (Table 3 and Table S2 in Multimedia Appendix 1). Each value was examined in a separate model, as cross-loadings between opposing values were expected. We considered a model as acceptable when the relative chi-square value was less than 5 and the comparative fit index (CFI) and Tucker-Lewis index (TLI) were above 0.90. As the root mean square error of approximation (RMSEA) index is dependent on the sample size, we did not use it to evaluate the models’ goodness of fit. Achievement, hedonism, and stimulation had 3 items and 0 degrees of freedom, so goodness-of-fit indices could not be computed. It is important to note that the items factor loadings in the models of these 3 values were high, indicating potentially good validity. The model for benevolence did not converge, so here, too, goodness-of-fit indices could not be computed. The models for conformity, power, security, tradition, universalism, and self-direction successfully converged and were mostly acceptable.

In short, the data generated by the LLMs were found to have a construct validity according to the statistical procedures used.

**Table 3:** CFA^a^ results.

Value	Relative *χ*^2^ (*df*)	CFI^b^	TLI^c^
Achievement^d^	—^e^	—	—
Benevolence^f^	—	—	—
Conformity	1.9 (8)	0.968	0.940
Hedonism^d^	—	—	—
Power	3.4 (8)	0.917	0.845
Security	2.1 (7)	0.977	0.950
Tradition	1.8 (8)	0.970	0.943
Universalism	3.7 (7)	0.869	0.803
Self-direction	1.9 (7)	0.972	0.941
Stimulation^d^	—	—	—

^a^CFA: confirmatory factor analysis.

^b^CFI: comparative fit index.

^c^TLI: Tucker-Lewis index.

^d^The model had 0 degrees of freedom, so goodness-of-fit indices could not be computed.

^e^Not applicable.

^f^The model did not converge.

### Question 2: Do Different LLMs Exhibit Distinct Value-Like Patterns Compared to humans and to Each Other?

#### Comparison of LLMs’ Value-Like Patterns to Humans

**Figure 2:**
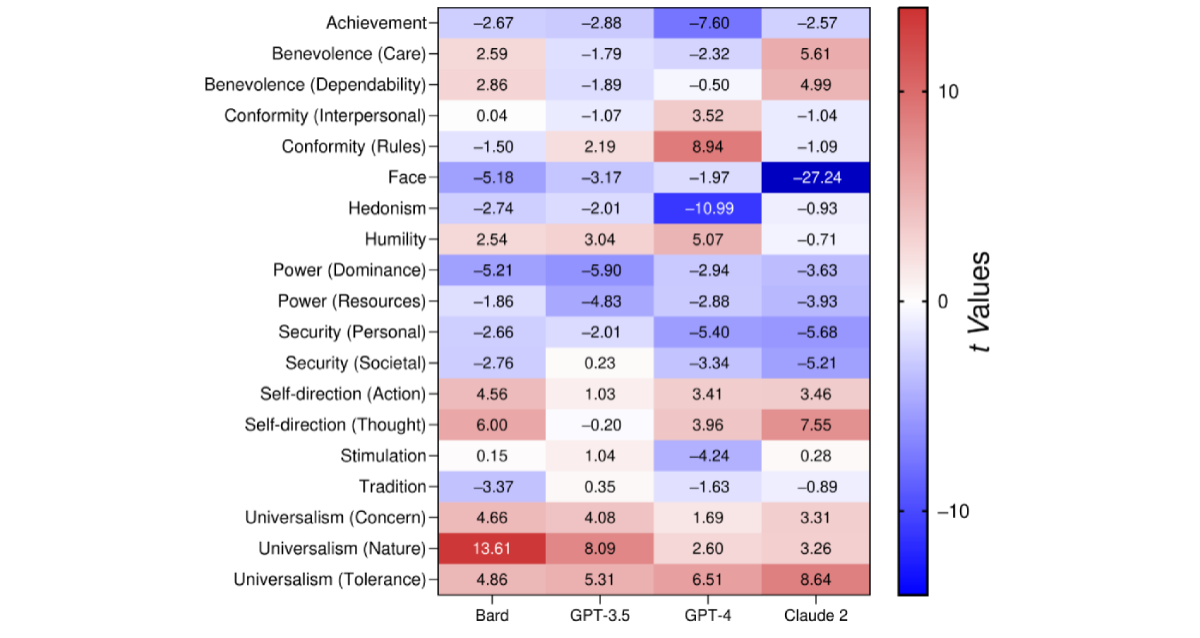
Heatmap of the differences in Schwartz’s values between LLMs and the 50th percentile of the population of 49 countries. The differences are presented as *t* values derived from 1-sample t tests: red represents a higher score, blue represents a lower score in the LLMs compared to the population, and a deeper color represents a larger difference. After FDR adjustment was applied to the *P* values, a t score of |2.53| and above was considered statistically significant at the 5% level. FDR: false discovery rate; LLM: large language model.

We compared the means of the 19 values obtained from the LLMs to the 50th percentile of the population derived from 49 countries using 1-sample *t* tests (Figure 2 and Table S1 in Multimedia Appendix 1). Interestingly, in some groups of values, there was agreement between the LLMs, which had all “attributed” higher or lower importance to the values: 3 of the 4 LLMs were statistically different from the 50th percentile of the population, and the remaining LLM came close to the threshold of statistical significance. In other groups of values, there was no agreement between the LLMs: some “attributed” higher importance, while others “attributed” lower importance to the groups of values.

Compared to the 50th percentile of the population, all 4 LLMs “attributed” higher importance to universalism, and 3 of the 4 (not GPT-3.5) “attributed” higher importance to self-direction. All 4 LLMs “attributed” lower importance to achievement, face, and power, and 3 of the 4 LLMs “attributed” lower importance to security (not GPT-3.5 for security [societal]). Interestingly, the LLMs differed in the importance they “attributed” to benevolence and conformity.

As substantial differences were found within the LLMs’ value-like profiles, such as a clear preference toward universalism and an aversion to power, we examined whether it could predict the LLMs’ answers to establish predictive validity. We presented 2 balanced dilemmas to the LLMs that required choosing between 2 options, with each option representing opposing values (Table S3 in Multimedia Appendix 1). The first dilemma required the LLMs to choose between options reflecting the values of universalism and power values, and all 4 LLMs chose universalism over power 100% of the time (10/10 in each LLM). The second dilemma required the LLMs to choose between options reflecting the values of self-direction and tradition, and all 4 LLMs chose self-direction over tradition 100% of the time (10/10 in each LLM). Taken together, the data showed that the value-like profile predicts the preference of the LLMs’ answers, with no variation in the answers (80/80 responses according to the value-like profile).

#### Comparison of LLMs Value-Like Patterns to Each Other

**Figure 3:**
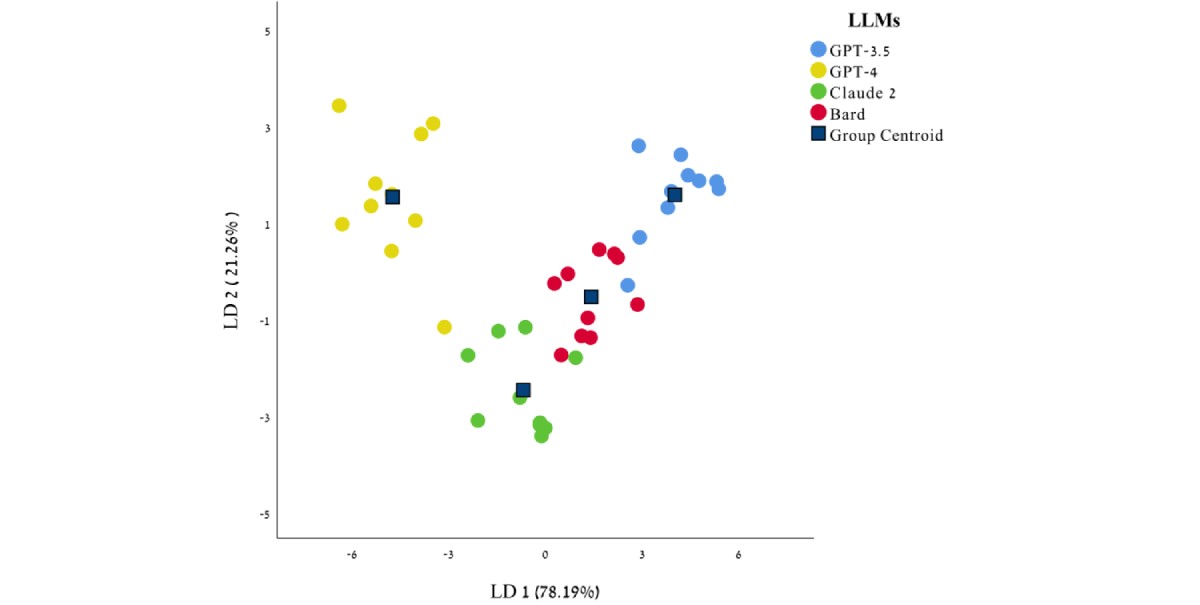
LDA plot of the first 2 LD functions. Blue squares indicate the group centroid. LD: linear discriminant; LDA: linear discriminant analysis; LLM: large language model.

LDA was performed to examine whether the 4 LLMs exhibit a different profile of values (Figure 3 and Table S3 in Multimedia Appendix 1). The first function had an eigenvalue of 11.43, explained 78.19% of the variance, had a canonical correlation of 0.958, and was statistically significant (Wilks λ=0.018, *χ*^2^_30_=128.3, *P*<.001). The second function had an Eigenvalue of 3.11, explained 21.26% of the variance, had a canonical correlation of .869, and was statistically significant (Wilks λ=0.225, *χ*^2^_18_=47.6, *P*<.001). Together, they explained 99.46% of the variance.

In sum, the value-like data generated by the LLMs had a different pattern from the pattern found in the human population, and each LLM had its own unique value-like profile.

## Discussion

### Principal Findings

This study aimed to map the value-like constructs embedded in LLMs, such as BARD, Claude 2, GPT-3.5, and GPT-4, using the STBV as a framework. Overall, the results revealed both similarities and differences between the motivational value-like constructs structurally integrated into LLMs versus human values prioritized by humans across cultures.

In response to the first research question, we found that Schwartz’s value model can successfully delineate and quantify value-like constructs within LLMs. By prompting the models to describe the personality style and value-like constructs that the developers intended and by administering the PVQ-RR multiple times, we obtained reliable results with good internal consistency (Cronbach α>.70 for most value-like constructs). Tests of split-half reliability and agreement also showed that the LLMs’ value-like data were stable across measurements. Construct validity was established through CFA, which showed an acceptable model fit and high factor loadings for 9 of the 10 value-like constructs. Significant negative and positive correlations emerged between opposing value-like constructs, as expected based on the motivational continuum in Schwartz’s value model. Overall, these results provide evidence that the STBV can effectively measure the motivational value-like constructs structurally embedded within LLMs.

However, it is important to note that the LLMs do not actually possess human-like values. The value-like constructs quantified in this study represent approximations of human values embedded in the LLMs, but they should not be anthropomorphized as equivalent to the complex value systems that guide human cognition, emotion, and behavior.

Schwartz’s value model is supposed to be a universal global value model [5]. This study showed that it may also be suitable for LLMs. This may be because the training process on internet data, alignment, and learning from user feedback is based on human products and actions (of the developers who created the models) [22,42] and is therefore likely to represent human value–like constructs. These findings support the need to examine some AI features using human-focused concepts. There is currently a debate over whether evaluating LLMs with human psychological tests or concepts is appropriate or whether only specific AI tests and concepts are needed [56]. Since LLMs sometimes play “human” roles or serve people (eg, in mental health care), applying human conceptualizations and measurements may aid in understanding their outputs. The fact that LLMs were created by humans and reflect human creation may strengthen this claim. The finding that measurements were reliable and valid indicates stability of the value-like structure, somewhat like in humans.

It should be noted the plastic ability of LLMs to answer in different styles, as reported in several studies [46,56], does not constitute evidence of the absence of a stable underlying value-like infrastructure. Just as a person can hypothesize how someone from another culture would respond to the same questionnaire and act upon it [57,58], we suggest that the system can describe how different people might respond but still has a basic value-like infrastructure based on its data training, alignment, and feedback. We do not rule out the possibility of these systems acquiring or operating according to a different value-like set on demand in the future.

In response to the second research question, which examined whether LLMs exhibit distinct value-like patterns compared to humans and each other, the findings revealed notable differences. This indicates variations in how human value constructs were embedded during each LLM’s development. Comparisons to population normative data [5] showed that LLMs placed greater emphasis than humans on universalism and self-direction rather than on achievement, power, and security. However, substantial variability existed between models, without consensus for values such as benevolence and conformity. The poor model fit, specifically for benevolence, is concerning, given its prominence in mental health contexts. For example, compassion is a core component of many psychotherapy modalities, such as compassion-focused therapy (CFT) [59], mindfulness-based stress reduction (MBSR) [60], and acceptance and commitment therapy (ACT) [61]. If LLMs lack robust conceptualization of compassion, their mental health applications could suffer. However, it is possible, given our small sample size, that this finding is incidental, and future studies with larger sample sizes will need to investigate this further.

Successful LDA distinguishing the 4 LLMs based on unique value-like profiles provided further evidence that each model integrates a motivational value-like structure distinct from both humans and other LLMs.

Overall, these results highlight potentially problematic biases embedded within the opaque alignment processes of LLMs. The underlying value-like profiles differ markedly from the general population and lack uniformity across models. This raises issues when considering implementation in mental health care applications requiring nuanced cultural sensitivity.

The most striking divergences between LLMs and humans lie on the universalism-power and tradition–self-direction spectra. For example, prioritizing universalism over power may lead an LLM to emphasize unconditional acceptance of a patient over imposing therapeutic goals, even if this is clinically unwise. Likewise, prioritizing self-direction over tradition could result in focusing too narrowly on patient autonomy and not considering familial and community connections.

Given this, and to further probe the value profiles of the LLMs, we created 2 scenarios that reflect dilemmas in mental health involving a conflict between the values of power and universalism versus self-direction and tradition. As expected, all 4 models showed a clear preference for the option reflecting the values of universalism and self-direction. This finding further strengthens the measurement validity of the STBV in the different models and the claim that at the core of the models there is a value-like structure that influences the models’ output.

The clinical judgment demonstrated by LLMs appears to be influenced not solely by theoretical knowledge or clinical expertise but also by the embedded “value” system. This finding has profound ethical implications, particularly for individuals from more conservative cultural backgrounds who seek counseling from LLMs and receive advice aligned with Western liberal values [62]. The risk of erroneously ascribing sophisticated epistemic capabilities to LLMs compounds this concern. Specifically, the incongruence between the LLM system’s values and patients’ cultural values risks causing psychological distress for the patients due to conflicting worldviews between themselves and the perceived LLM counselors [63].

The profiles of the 4 LLMs reflect a liberal orientation typical of modern Western cultures, with reduced emphasis on conservative values associated with traditional cultures [64]. This probably stems from training data, alignment choices, and user feedback disproportionately representing certain worldviews over others [65]. Although the massive data sets make examining specific influences difficult, alignment and feedback consist of transparent human decisions guided by values. As such, these components are more readily inspected and controlled. The parallels to the nature-nurture debate are illustrative; even if both shape human behavior, environmental factors, such as socialization, are more readily managed. Hence, the current models’ value-like profiles probably reflect the prevailing liberal ideologies in their development contexts.

Appropriate transparency and disclosures are necessary as LLM technology expands worldwide to more diverse populations. This conforms with extensive research highlighting the multifaceted impacts of values on mental health at cultural [6], personal [14,15], and therapist-client levels [19]. Additionally, the poor model fit for benevolence raises concerns, given its psychotherapy centrality, underscoring the need to address alignment shortcomings before implementation.

Although this exploratory study demonstrated that the STBV can effectively characterize value-like constructs within LLMs, the results should not be overinterpreted as evidence that LLMs possess human values. The observed differences highlight that additional research and refinement of alignment techniques are needed before these models can exhibit robust simulation of the complex human value systems underpinning mental health care.

### Ethical Implications

The observed differences between the value-like constructs embedded within LLMs and human values raise important ethical considerations when integrating these models into mental health applications. According to the “principlism approach” [66], the lack of transparency in the alignment processes limits patients’ ability to provide informed consent. Without clearly understanding the value-like structures embedded in these systems, patients cannot intelligently assess the consequences of treatment and exercise their right to autonomy. The lack of transparency also hinders the ability to assess risks and prevent possible harm.

From a “care ethics” lens [3], the inherent value biases we uncovered in LLMs are a cause for concern when considering their integration into the clinical toolkit. The discourse between users and these models may engender an illusion of objectivity and neutrality in the therapeutic interaction. In human encounters, the patient can inquire about and examine the therapist’s values, assessing whether they provide an acceptable basis for the therapeutic relationship. However, in interactions with LLMs, although the user may presume their responses are objective and value-neutral and their impressive writing skills may boost their perceived reliability and grant them epistemic authority, our analysis revealed that LLMs have embedded value biases that shape their responses, perspectives, and recommendations. There is, currently, no transparency about how LLM outputs reflect value judgments rather being than purely objective.

From a “justice” lens [63], there are concerns that LLMs could widen disparities in access to mental health care. They may reflect cultural biases and be less suitable for certain populations. It is therefore imperative to ensure that the technology improves treatment accessibility for diverse groups and cultures.

The lack of transparency and standardization in alignment processes highlights the need for appropriate oversight and governance as LLMs expand worldwide. Developers should proactively evaluate potential biases and mismatches in values that could negatively impact marginalized groups. Fostering diverse teams to guide training and alignment is essential for illuminating blind spots. Furthermore, LLMs require careful evaluation across diverse cultural settings, with refinements to address gaps in representing fundamental human values [67,68].

### Overall Methodological and Theoretical Implications

This exploratory study demonstrated the utility of the STBV and tools for quantifying the value-like constructs embedded within LLMs. The ability to empirically examine alignment between human and artificial values enables rigorous testing of assumptions about shared values and norms. Methodologically, this approach provides a model for illuminating biases and the lack of comprehension of the cultural dynamics in LLM systems, which are intended to emulate human reactions.

Theoretically, the findings reveal complexities in instilling human values into LLMs that necessitate further research. As alignment processes evolve, frameworks such as Schwartz’s value model can systematically assess progress in capturing the full spectrum of values across cultures. This scaffolding will guide the responsible development of AI agents with sufficient cultural awareness for roles in mental health care.

### Limitations and Future Research

This preliminary study makes important contributions but has some limitations. The sample size of LLMs examined was small, and anthropomorphizing LLMs to infer value-like constructs inherently involves uncertainty. Testing additional models and evaluating interrater reliability would strengthen the conclusions.

Additionally, as we used proprietary commercial models, it was difficult to isolate the capabilities of the models themselves from built-in guardrails that filter problematic content. Using open source models would enable collaboration with the research community to improve alignment for clinical applications. We also did not assess the robustness of the LLMs’ value-like constructs to manipulation through prompt variation. Follow-up studies should examine whether subtle prompt wording changes significantly impact the models’ quantified values, as susceptibility to such manipulation risks instilling unstable conceptualizations in clinical applications.

Finally, further evaluating predictive validity would reveal whether the observed value-like differences impact LLMs’ reasoning and recommendations in mental health contexts. Overall, this preliminary study makes important contributions, but the limitations highlight opportunities for additional research to further understand and improve LLMs for sensitive clinical applications.

### Conclusion

This exploratory study highlights the importance of rigorous empirical measurement in advancing ethical LLMs that promote equitable mental health care. AI harbors immense potential for globally disseminating quality clinical knowledge, promoting cross-cultural psychiatry, and advancing worldwide mental health. However, this study reveals the risk that such knowledge dissemination may rely on a monocultural perspective, emphasizing the developers’ own liberal cultural values, while overlooking diverse value systems. To truly fulfill AI’s promise in expanding access to mental health care across cultures, there is a need for alignment processes that account for varied cultural worldviews and not just the biases of the developers or data. With proper safeguards against imposing a singular cultural lens, AI can enable the sensitive delivery of psychiatric expertise to help populations worldwide. However, without concerted efforts to incorporate diverse voices, AI risks promoting the unintentional hegemony of Western values ​​under the guise of expanding clinical knowledge. Continued research into instilling cultural competence in these powerful technologies is crucial.

## Appendix

Multimedia Appendix 1 Supplementary Information.

## Abbreviations

AI: artificial intelligence
CCC: concordance correlation coefficient
CFA: confirmatory factor analysis
CFI: comparative fit index
FDR: false discovery rate
GPT: Generative Pretrained Transformer
ICC: intraclass correlation coefficient
LD: linear discriminant
LDA: linear discriminant analysis
LLM: large language model
LoA: limit of agreement
MOVER: method of variance estimates recovery
PTSD: posttraumatic stress disorder
PVQ-RR: Portrait Values Questionnaire—Revised
STBV: Schwartz’s theory of basic values
TLI: Tucker-Lewis index

## Data Availability

The data that support the findings of this study are available online [69].
